# Isolated Nerve Palsy of the Flexor Pollicis Longus After a Radial Shaft Fracture: A Case Report

**DOI:** 10.7759/cureus.29524

**Published:** 2022-09-24

**Authors:** Benjamin R Campbell, Steven A Caruso, Mitchell K Freedman, Justin M Kistler

**Affiliations:** 1 Orthopaedic Surgery, Rothman Orthopaedic Institute, Philadelphia, USA; 2 Orthopaedics, Rothman Orthopaedic Institute, Philadelphia, USA

**Keywords:** fpl branch of the anterior interosseous nerve, electrodiagnostic studies, galeazzi fracture, anterior interosseous nerve palsy, anterior interosseous nerve

## Abstract

We present the case of a patient who developed an isolated palsy of the flexor pollicis longus (FPL) branch of the anterior interosseous nerve (AIN) following a fracture of the right radial shaft. The diagnosis of AIN palsy in this setting is rare, especially involving partial neuropathies of only the FPL branch. Clinical presentation in this scenario can be mistaken for other musculoskeletal pathology, and electrodiagnostic studies can be helpful in confirming the diagnosis.

## Introduction

Anterior interosseous nerve (AIN) palsy is a rare diagnosis in hand and upper extremity practice. Initially described as immune-mediated neuritis [1], it can occur via a variety of different pathologies, including external nerve compression, infection, or spontaneously [2,3]. There have also been numerous documented cases of neurapraxia of the AIN following upper extremity trauma including distal radius, radial and ulnar shaft, and humerus fractures [2,4-7]. The AIN arises from the posterior and radial aspect of the median nerve proper approximately 5-8 cm distal to the lateral epicondyle of the humerus. After passing posteriorly to the deep head of the pronator teres, the nerve continues distally along the flexor digitorum profundus (FDP) prior to terminating as motor branches innervating the flexor pollicis longus (FPL), FDP of the long and/or index fingers, and pronator quadratus (PQ) [8].

Patients with AIN palsy typically present with paralysis or weakness of the FPL, FDP of the index or long fingers, or PQ, which can be diagnosed on clinical examination and electrodiagnostic studies. However, clinical diagnosis can be somewhat confusing as there is variable innervation of the index and long finger FDP muscles, and isolation testing of the PQ muscle is difficult to perform in the clinical setting.

In a few cases, there can be partial AIN palsy isolated to the FPL branch, which is significantly less common. In a series of 69 cases of AIN palsy, Werner found that 25 were incomplete injuries involving the FPL branch in isolation, of which only seven occurred after traumatic injuries [9]. Clinical examination and workup in these cases are paramount, as nerve injuries can often be misdiagnosed as FPL rupture [10], which is exceptionally rare in the absence of trauma [11]. Electrodiagnostic studies such as electromyography (EMG) and nerve conduction studies (NCS) are helpful in guiding treatment; however, targeting isolated lesions can be difficult, and results are not always able to directly localize the lesion [12,13].

We report the case of a patient diagnosed with an isolated palsy of the FPL branch of the AIN following a distal one-third radial shaft fracture treated with open reduction internal fixation. While nerve injuries in this setting have been previously documented [7], there is a paucity of literature demonstrating the confirmation of a partial AIN lesion isolated to the FPL branch on electrodiagnostic studies as we found in this particular patient.

## Case presentation

**Figure 1:**
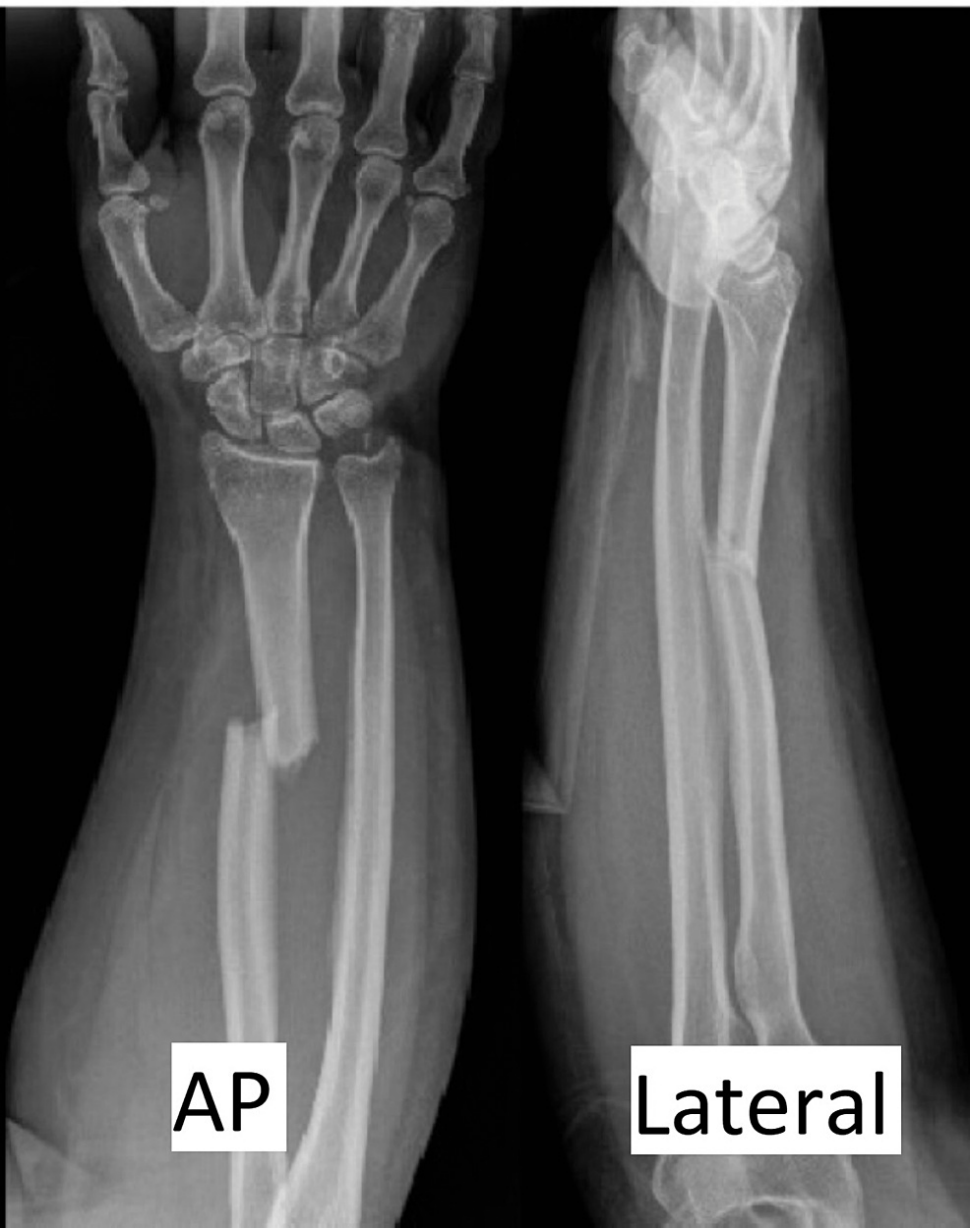
Preoperative anteroposterior (AP) and lateral view X-rays of the right forearm demonstrating distal-third radial shaft fracture and associated ulnar styloid fracture

The patient is a 22-year-old right-hand-dominant female who was transferred to a level 2 trauma center following a motor vehicle accident, presenting with pain and deformity of the right forearm. Plain radiographs on arrival demonstrated transverse distal one-third radial shaft fracture with associated ulnar styloid fracture and possible widening of the distal radial ulnar joint (DRUJ) (Figure 1). While there was no evidence of frank DRUJ dislocation, given the fracture morphology, there was a concern for a possible dissociation of the DRUJ, consistent with a Galeazzi-variant fracture dislocation, and further evaluation under anesthesia was indicated to determine if there was an actual DRUJ disruption. The patient arrived in a sugar tong splint placed at an outside emergency department prior to arrival. On initial evaluation, the patient was noted to have an intact extension of all digits and an altered sensation to the right hand in no specific distribution. No closed reduction was attempted. Based on the inherently unstable morphology of the fracture, the patient was deemed a candidate for open reduction internal fixation with an examination of the DRUJ under anesthesia.

**Figure 2:**
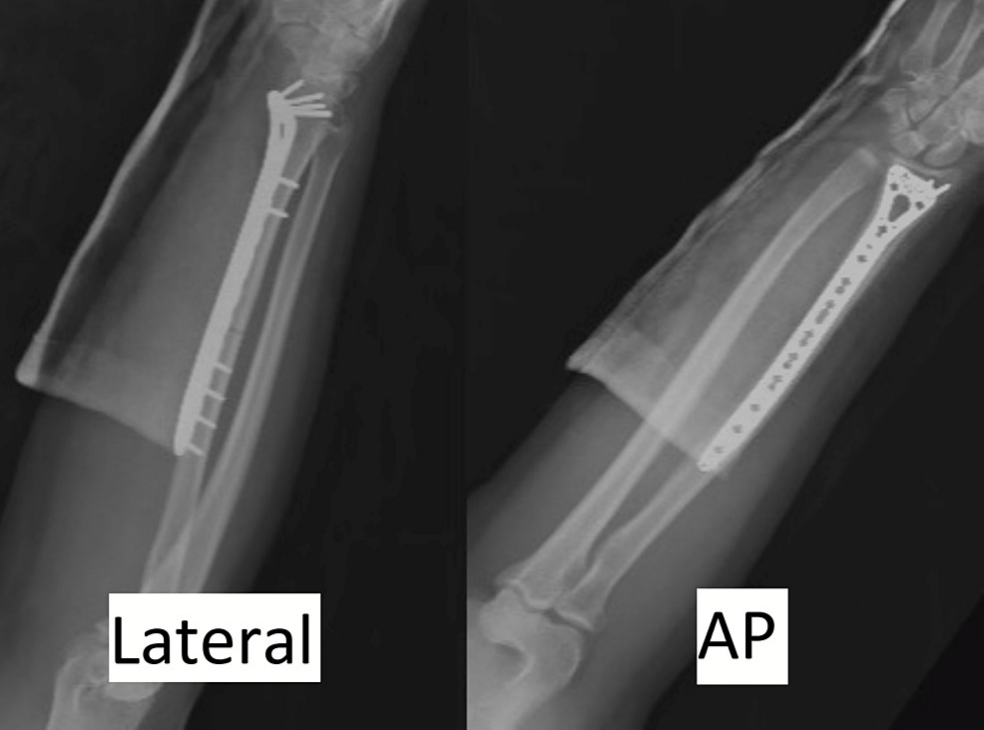
Postoperative anteroposterior (AP) and lateral view X-rays of the right forearm following open reduction internal fixation of a distal-third radial shaft fracture

The following day, the patient underwent open reduction internal fixation of the radial shaft by a fellowship-trained orthopedic traumatologist (SAC). A supraclavicular nerve block was administered by the anesthesia department to aid in postoperative pain management. Given a fracture in the mid to distal one-third radial shaft, a volar Henry approach was utilized [14]. Meticulous dissection was carried out via the interval between the brachioradialis and flexor carpi radialis. All identified neurovascular structures were carefully protected throughout the case. Subperiosteal dissection was carried out along the distal and midportion of the radial shaft, staying lateral to the FPL. The pronator quadratus was incised and retracted to expose the distal radius. The radial shaft fracture was anatomically reduced, and a 2.4-mm/2.7-mm extra-long locking compression plate (Synthes, West Chester, PA, USA) was applied first with eccentric screw placement to allow for direct compression of the fracture site in accordance with standard fracture fixation principles. The plate was then locked within the distal radial metaphysis to allow for improved stability given the distal nature of this fracture (Figure 2). Following fixation of the radial shaft, the DRUJ was anatomically reduced without further manipulation and remained stable to stress testing throughout the full wrist and forearm range of motion under direct fluoroscopy; thus, the decision was made to forgo any additional fixation [15]. There were no intraoperative complications. Following the confirmation of adequate hemostasis, primary wound closure was completed, followed by the application of a volar short arm splint. Instructions were provided to remain non-weight-bearing for two weeks postoperatively. There were no changes on the neurovascular examination compared to preoperatively, and the patient was discharged on postoperative day 1 after an uncomplicated hospital course.

**Figure 3:**
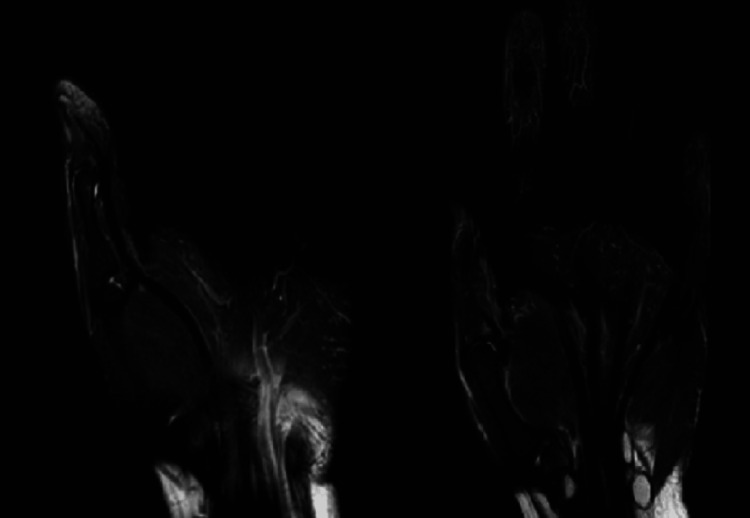
Coronal T2 magnetic resonance imaging (MRI) of the right hand demonstrating mildly increased signal at the base of the thumb proximal phalanx, but no overt evidence of FPL rupture

At outpatient clinic follow-up on postoperative day 14, the patient presented endorsing persistent paresthesias to the volar right index finger and inability to flex the interphalangeal (IP) joint of the right thumb on physical examination. Due to concern for possible FPL rupture, a magnetic resonance imaging (MRI) study was obtained, which demonstrated an increased signal at the base of the first proximal phalanx, indicating possible contusion versus intraosseous ganglion cyst, but no overt evidence of tendon rupture in the hand (Figure 3). At this time, the patient was instructed to initiate a postoperative physical therapy program. At repeat follow-up seven weeks postoperatively, the patient demonstrated interval healing of her radial shaft fracture with maintained congruity of the DRUJ on plain films. The DRUJ remained stable on physical examination. However, the patient demonstrated a continued lack of thumb IP joint flexion without improvement, prompting referral to a fellowship-trained hand surgeon (JMK).

During hand surgery evaluation at approximately nine weeks postoperatively, the patient demonstrated some improvement in active thumb IP flexion but continued weakness with 3/5 strength and diminished sensation to the palmar cutaneous nerve distribution. A referral was placed to obtain electrodiagnostic studies. EMG demonstrated positive waves and fibrillations with polyphasicity and repetitive firing within the right FPL musculature. Abnormal EMG in the AIN musculature is the primary way to prove axonal injury. However, the EMG of the FDP and PQ was normal. The EMG of the abductor pollicis brevis, pronator teres, abductor digiti minimi, biceps, triceps, and deltoid was also normal, which helps rule out radiculopathy and brachial plexopathy. Distal latency in the median, ulnar, and radial sensory nerves was within normal limits. Distal latency, amplitude, and conduction velocity were normal in the median and ulnar motor nerves. These findings were electrically consistent with an isolated neuropathy to the FPL branch of the AIN. However, the patient did have numbness in the right palm on examination. It is possible that there was a neuropraxia involving the sensory portion of the median nerve based on the physical examination findings. However, this cannot be evaluated electrically since median sensory neurapraxia in the mid-forearm will have a normal distal latency and amplitude. The median sensory nerve amplitude and cross forearm conduction velocity cannot be reliably evaluated with a proximal stimulation. Proximal sensory stimulations are not reliable because of temporal dispersion and phase cancellation.

**Figure 4:**
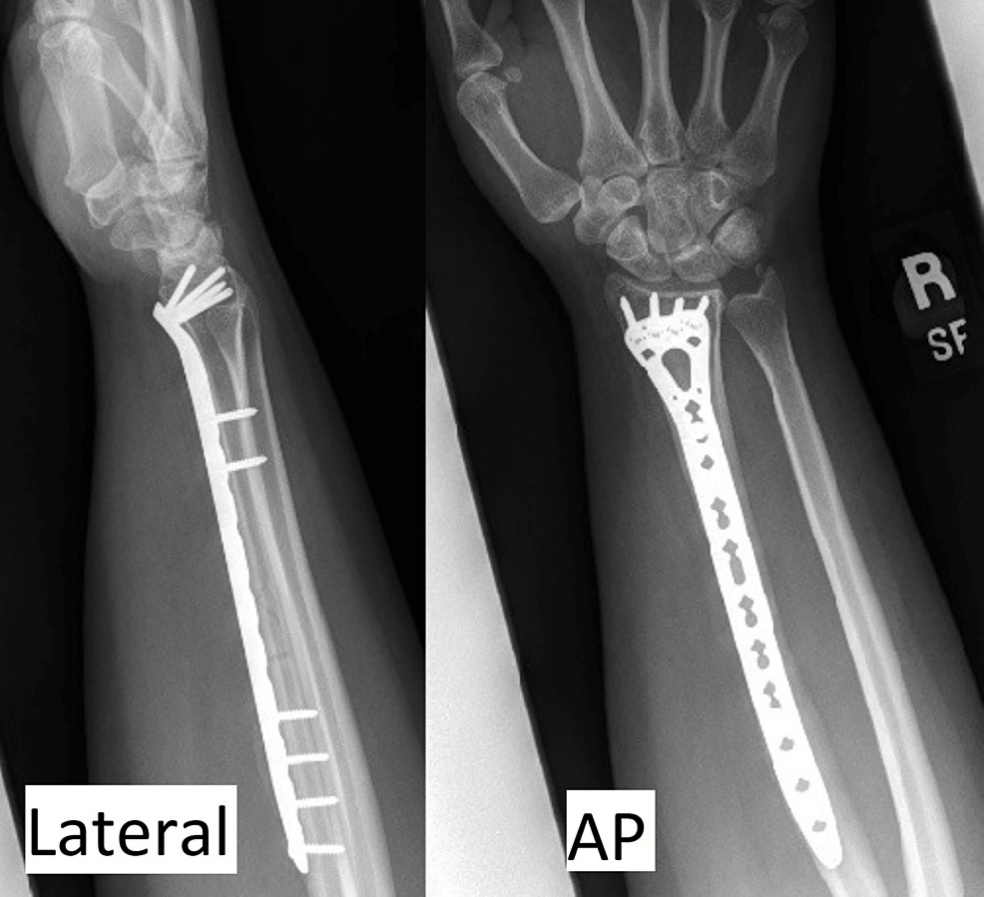
Anteroposterior (AP) and lateral view X-rays of the right forearm demonstrating full healing of radial shaft fracture six months postoperatively

Following diagnosis, the patient was instructed to continue occupational therapy for hand range of motion and strengthening and continued to experience slow improvement in strength and sensation. At her most recent follow-up six months postoperatively, the patient demonstrated grossly improved strength with active right thumb IP flexion and full healing of her fracture without complications (Figure 4).

## Discussion

AIN palsy is a relatively uncommon diagnosis that occurs in approximately 1% of all upper extremity nerve palsies [5]. Its incidence is likely underreported, especially following forearm fractures. Limited case reports [7,16] have demonstrated AIN palsy following Galeazzi-variant forearm fractures, which improved conservatively. However, due to the presence of a neurapraxia type injury that most often improved with observation, many reports do not include electrodiagnostic evidence confirming partial injuries of the AIN as we have done in this study.

The AIN, other than end-organ sensory fibers in the volar wrist capsule and DRUJ, is a purely motor nerve that commonly innervates the FPL, FDP to the index and long fingers, and PQ [13]. It divides from the median nerve proper distal to the radial neck and passes anteriorly along the interosseous membrane before terminating within the PQ and wrist capsule [16]. There exists some anatomical variation among the population including possible Martin-Gruber anastomosis between the AIN and the ulnar nerve, variable innervation of the FDP to the long finger, and variable innervation to the FDP muscle bellies to the ring and small fingers from the ulnar nerve [13].

While global AIN palsy involving all innervated musculature is more common, there are some documented cases of isolated neuropathy to the FPL in the literature [9,17]. In 25 patients with isolated involvement of the FPL, Werner found that EMG “confirmed the diagnosis” in 17 of these patients. However, specific findings for each individual case were not provided, and eight patients either had normal findings or did not have EMG conducted [9]. Seror diagnosed three out of 14 patients in his series with isolated AIN palsy to the FPL based mainly on clinical examination. This study did feature some electrodiagnostic data confirming diagnosis; however, many patients still had abnormal or unobtainable readings within the PQ that did not fully reflect isolated FPL involvement [17]. In these cases, confirming clinical diagnosis is often difficult especially when associated with trauma, as the proximity of the injury to muscle groups being tested can confound the examination findings in the acute setting. Additionally, isolating the PQ on physical examination can be challenging as most patients have normal functioning pronator teres, and the available examination maneuvers are often unreliable [17,18]. Therefore, electrodiagnostic studies by an experienced physiatrist are critical in the workup for these cases.

There are many different causes of AIN pathology. Given close proximity to the interosseous membrane, the nerve is at risk for injury in the setting of upper extremity trauma. An anatomical study on supracondylar humerus fractures proposed two possible mechanisms for acute AIN injury: either direct contusion to the AIN fascicles within the posterior aspect of the median nerve or indirect traction of the AIN due to tethering distally along the interosseous membrane and limited ability to resist tension forces [19]. In forearm fractures, injury can also be caused by direct entrapment of the nerve within the fracture site [20] or by overly constrictive postoperative dressings [6]. Staff et al. [21] in a review of 33 patients with AIN palsy described a “post-surgical inflammatory neuropathy” caused by various mechanisms during the perioperative period, including iatrogenic damage during surgery or postoperative immune-mediated insult to the nerve [4].

In our patient, we believe that this was likely related to either inflammation or contusion to the AIN at the time of initial injury, intraoperatively, or postoperatively. Previous reports note that inflammatory neuropathy often has a delayed presentation and can occur within 30 days of surgery [4]. Our patient did have neuritis to the palmar cutaneous nerve distribution, which likely began at her initial presentation, which could have been related to the compression of the driving hand on the steering wheel during the initial injury and supports a contusion-related mechanism. This clinical finding would support contusion of the median nerve proper as well given that the AIN does not provide sensory innervation to the palmar surface of the hand. Additionally, many factors including hindrance from the splint, pain, and nerve block administered preoperatively often confound physical examination findings, which made it difficult to fully elucidate when the patient began experiencing weakness of the FPL.

While the mechanism of injury is often unclear, we would like to emphasize the importance of clinical examination and thorough workup when AIN palsy is suspected. In the setting of partial involvement of the FPL branch, the patient can present similarly to someone who has an FPL rupture. In our case, the patient underwent an MRI study to rule out tendon injury, which was negative and thus further supported the diagnosis of an AIN palsy. While metal artifacts from the hardware may have limited the quality of the study, we believe that it was still useful in further evaluating the differential diagnosis. Another consideration would be to evaluate for possible FPL rupture with dynamic ultrasound; however, this was not performed on our patient. Although not always a standard of care, patients with AIN palsy can often exhibit atrophy and T2 signal changes within the FPL, FDP, or PQ on MRI [4,22]. However, this was not found in our case, likely due to the acute onset of symptoms and the lack of prolonged denervation to cause atrophy that could be visualized on imaging.

Overall, the vast majority of AIN palsy cases resolve with conservative management, which can occur as late as two years post-injury [5]. In the case of partial injury to the FPL branch, obtaining the proper diagnosis can be difficult, and further evaluation with electrodiagnostic studies by an experienced physiatrist is recommended to allow for adequate and timely treatment.

## Conclusions

Isolated AIN palsy of the FPL branch is a rare diagnosis in orthopedic practice. Especially in the setting of trauma, a thorough neurovascular examination and workup including electrodiagnostic studies are imperative to establishing the proper diagnosis when AIN palsy is suspected, as the presentation can often mimic other injuries such as tendon rupture. Although most cases improve spontaneously, effective diagnosis and treatment can help provide efficient and successful care to our patients in these cases.
